# S3QL: A distributed domain specific language for controlled semantic integration of life sciences data

**DOI:** 10.1186/1471-2105-12-285

**Published:** 2011-07-14

**Authors:** Helena F Deus, Miriã C Correa, Romesh Stanislaus, Maria Miragaia, Wolfgang Maass, Hermínia de Lencastre, Ronan Fox, Jonas S Almeida

**Affiliations:** 1 Digital Enterprise Research Institute, National University of Ireland at Galway, IDA Business Park, Lower Dangan, Galway, Ireland; 2 Biomathematics, Instituto de Tecnologia Química e Biológica, Universidade Nova de Lisboa, Av. da República, Estação Agronómica Nacional, 2780-157 Oeiras, Portugal; 3 Laboratório Nacional de Computação Ciêntifica, Av. Getúlio Vargas, 333,Quitandinha, 25651-075 Petrópolis, Brasil; 4 Sanofi Pasteur, 38 Sidney Street, Cambridge, MA 02139, USA; 5 Laboratory of Molecular Genetics, Instituto de Tecnologia Química e Biológica, Universidade Nova de Lisboa, Av. da República, Estação Agronómica Nacional, 2780-157 Oeiras, Portugal; 6 Research Center for Intelligent Media, Furtwangen University, Furtwangen, Germany; 7 Laboratory of Microbiology, The Rockefeller University, 10021 New York, USA; 8 Division of Informatics, Department of Pathology, University of Alabama at Birmingham, 619 South 19th Street, Birmingham, Alamaba, USA

**Keywords:** S3DB, Linked Data, KOS, RDF, SPARQL, knowledge organization system, policy

## Abstract

**Background:**

The value and usefulness of data increases when it is explicitly interlinked with related data. This is the core principle of Linked Data. For life sciences researchers, harnessing the power of Linked Data to improve biological discovery is still challenged by a need to keep pace with rapidly evolving domains and requirements for collaboration and control as well as with the reference semantic web ontologies and standards. Knowledge organization systems (KOSs) can provide an abstraction for publishing biological discoveries as Linked Data without complicating transactions with contextual minutia such as provenance and access control.

We have previously described the Simple Sloppy Semantic Database (S3DB) as an efficient model for creating knowledge organization systems using Linked Data best practices with explicit distinction between domain and instantiation and support for a permission control mechanism that automatically migrates between the two. In this report we present a domain specific language, the S3DB query language (S3QL), to operate on its underlying core model and facilitate management of Linked Data.

**Results:**

Reflecting the data driven nature of our approach, S3QL has been implemented as an application programming interface for S3DB systems hosting biomedical data, and its syntax was subsequently generalized beyond the S3DB core model. This achievement is illustrated with the assembly of an S3QL query to manage entities from the Simple Knowledge Organization System. The illustrative use cases include gastrointestinal clinical trials, genomic characterization of cancer by The Cancer Genome Atlas (TCGA) and molecular epidemiology of infectious diseases.

**Conclusions:**

S3QL was found to provide a convenient mechanism to represent context for interoperation between public and private datasets hosted at biomedical research institutions and linked data formalisms.

## Background

Knowledge engineering in the Life Sciences is challenged by the combination of high specificity and high heterogeneity of the data needed to represent and understand Biology's systemic puzzles. Despite the deluge of data that has invaded life sciences in the past decade [1], data-driven discovery in Biology is hindered by a lack of enough interlinked information to allow statistical algorithms to find the patterns that inform hypothesis-driven research [2,3]. Life Sciences research relies heavily on bioinformatics integration tools like Ensembl [4], the UCSC genome browser [5], Entrez Gene [6] or the gene ontology [7] because these offer researchers portals to a wealth of interlinked biological annotations within the context of their experimentally derived results, thus playing a lead role in advancing scientific discovery. The amount of time and effort required to develop and maintain such tools has prompted Linked Data approaches for data integration to become increasingly relevant in Health Care and Life Sciences (HCLS) domains [8-10]. Briefly stated, Linked Data can be described as a bottom-up solution for data integration: its focus is on creating a global Web of Data where typed links between data sources provide rich context and expressive reusable queries over aggregated and distributed heterogeneous datasets [8,11-13]. The architecture of that Web is expected by its original architects [14] to require a representation of usage contexts that can be applied in the collaboration and controlled sharing of data. When this functionality is supported, as that report anticipates, "social machines" will be able to manage the simultaneous and conflicting views of data that fuel scientific debate. The S3DB knowledge organization system was designed to provide baseline support for that bottom-up process by addressing a recurrent need for controlled sharing of HCLS datasets [15,16]. This report describes a convention, the S3QL language, to query and manipulate it. It will also be demonstrated that S3QL provides a convenient mechanism to engage Linked Data in general.

### 1.1 Linked Data Best Practices

Linked data best practices set the stage for an interlingua of relational data and logic in the web [17] by the definition of core principles that can be summarized as: 1) information resources should be identified with HTTP universal resource identifiers (URIs); 2) information should be served against a URI in a standard semantic web format such as the Resource Description Framework (RDF) and 3) links should be established to information resources elsewhere [10]. For large datasets, it is also convenient that a web service supporting SPARQL, the protocol and RDF query language, is also deployed [18]. Aggregation of data sources is available either by accessing metadata about the datasets as RDF [19,20], or through direct aggregation of RDF assertions in a single knowledgebase [21,22]. To ensure contextual consistency and reusability across datasets, data elements and descriptors are mapped using standard vocabularies, namespaces and ontologies [23-25].

### 1.2 Challenges involved in Publishing Primary Experimental Life Sciences Datasets as Linked Data

The value of linked data for life scientists lies primarily in the possibility to quickly discover information about proteins or genes of interest derived, for example, from a microarray or protein array experiment [26]. Life scientists involved in primary research still face significant challenges in harnessing the power of Linked Data to improve biological discovery. Part of the difficulty lies in the lack of adequate and user-friendly mechanisms to publish biological results as Linked Data prior to publication in scientific articles. Efforts in linking life sciences data typically focus on datasets which are already available in structured and annotated formats, i.e. after the researchers have analysed, correlated and manually annotated their results by browsing the literature or submitting their data to multiple web-based interfaces [27,28]. Current research [29,30] and our own experience in developing content management systems for health care and life sciences [26,31,32], has identified the need to go beyond those data sets by creating mechanisms for contextualizing linked life sciences data with attribution and version before it can be shared with a stable annotation. Advances have been made in that direction by other research efforts such as the recent publication of VoiD as a W3C note [33].

The technological advancements that will make primary Life Sciences experimental results an integral part of the Web of Data are also thwarted by challenges which go beyond infrastructure and standards [30]. In particular, HCLS datasets often include data elements, such as those that could be used to identify individual patients, with stringent requirements for privacy and protection [34]. The typical approach to privatizing data has been to make it the responsibility of the data providers. Although this may provide a temporary solution for a small number of self-contained datasets, it quickly becomes unmanageable when datasets aggregate both public and sensitive data from multiple sources, each with its own requirements for privacy and access control [35].

One final common concern in Life Sciences is the need to enable data experts to edit and augment the data representation models; failure to support this flexibility has lead in the past to misinterpretation of primary experimental data due to absence of critical contextual information [36,37].

### 1.3 Knowledge organization systems for Linked Data

In order to address the information management needs of Life Scientists, the practice of Linked Data standards must be coupled with the implementation of Knowledge Organization Systems (KOSs), a view also espoused by the W3C, where the Simple Knowledge Organization System (SKOS) has been recently proposed as a standard [38,39]. In previous work we proposed the design principles of a KOS, the Simple Sloppy Semantic Database (S3DB) [15,16,40]. The S3DB core model is, much like SKOS, task-independent and light-weight. Implementation of the S3DB Core Model and operators resulted in a prototype that has been validated and tested by Life Scientists to address pressing data management needs or, in particular, as a controlled Read-Write Linked Data system [31,41-43]. S3DB was shown to include the minimum set of features required to support the management of experimental and analytic results by Life Sciences experts while making use of Linked Data best practices such as HTTP URI, subject-predicate-object triples represented using RDF Schema, links to widely used ontologies suggested by NCBO ontology widgets [44] or new OWL classes created by the users and a SPARQL endpoint [41]. Although complying with these practices is enough to cover the immediate query or "read" requirements of a Linked Data KOS, we found that efficient data management or "write" operations, such as inserting, updating and deprecating data instances within a KOS could be more efficiently addressed with the identification of the S3DB Query Language (S3QL), a Domain Specific Language (DSL) devised to abstract most of the details involved in managing interlinked, contextualized, RDF statements.

S3QL is not meant as an alternative to SPARQL but rather as a complement: data management operations enabled by S3QL can also be formalized in SPARQL. However, the availability of a data management DSL that can be serialized to SPARQL provides an abstraction layer that can be intuitively used by domain experts. As such, DSLs can provide a solution for bridging the gap between the formalisms required by Linked Data best practices such as SPARQL and RDFS, and the basic controlled read/write management requirements of HCLS experts [12,45,46]. DSLs optimize beyond general purpose languages in the identification of the domain in which a task belongs, drastically reducing the development time [47]. The task of adding a graph to a triple store is supported by most graph stores by means of the SPARQL 1.1. Update language [48]. To enable controlled "write" operations targeting the dataset, it would be useful to annotate, for example, the creator of a named graph, under which circumstances it was created and who has permission to modify it. Similarly, upon changes to the dataset, annotation of the modifier and a comment describing the change would be in the interest of the communities using the data. Many triple stores are in fact quad stores to enable partial support of that requirement for contextual representation. The most common approach is to use a named graph, a set of triples identified by a URI [49] that indicates the source of a graph. The S3DB Query Language (S3QL) presented in this report was devised with the intent of automating Linked Data management by creating those contextual descriptors in a single S3QL transaction, including author, creation date and description of the data.

By making use of those contextual descriptors, we propose a method for fine grained permission control in S3QL that relies on *s3db:operators *[50], a class of functions, with states, that may be used as the predicate of an RDF triple between a user and a dataset with privacy requirements. These operators, described in [40] and made available for experimentation at [51], operate on the adjacency matrix defined by the nodes and edges of an RDF graph. They can be applied in a variety of scenarios such as optimizing queries or, as is the case with S3QL, to propagate permission assignments. In the latter case, an adjacency matrix includes both the edges between instances of S3DB entities and the transitions of permission on S3DB entities such as, e.g. the assertion that a User's permission on a Project propagates to its entities. Accordingly, by defining user permissions as states of *s3db:operators*, the core model's adjacency matrix is used to propagate the ability to control, view and modify S3DB entities.

We have found the target audience for S3QL to be both life sciences application developers, who use it through a RESTful application programming interface (API), and life sciences researchers who use it through user interfaces for weaving the ontologies that best represent the critical contextual information in their experimental results. The applicability of S3QL to other linked data KOSs such as the Simple Knowledge Organization System (SKOS) [39] is explored with an example and the advantages of the solution proposed are discussed in three biomedical datasets with very different requirements for controlled operations: gastrointestinal clinical trials [42], cancer genomic characterization [41] and molecular epidemiology [52].

## Methods

This section overviews the core model for S3DB, including the set of operators that enable fine grained permission control and the distributed infrastructure supporting S3QL. The principles defined here are implemented as a prototypical application available at http://s3db.org.

### The S3DB Knowledge Organization Model

S3QL is a DSL to programmatically manipulate data as instances of entities defined in a KOS. One of the key features of the KOS defined using the S3DB core model [16] is the use of typed named graphs to separate the identification of the domain, the metadata describing the data, from its observational instantiation - the data itself. We have previously shown that this approach to representing RDF greatly facilitates the assembly of SPARQL and lowers the entry barrier for biomedical researchers interested in using Semantic Web Technologies to address their data management needs [41]. That separation is achieved by using the representation of domain as triples that are themselves the predicates of the statements that instantiate that domain (as detailed by Fig. two in [40]). For example, the triple *[Person hasAge Age]*, identified as *:R12 *through a named graph of type *s3db:rule*, describes the domain while the triple *[John: R12 26 ]*, identified by a named graph of type *s3db:statement*, instantiates that domain. Through the logic encoded in the RDF Schema definition of domain (rdfs:domain) and range (rdfs:range), the assertion that "John" is of type "Person" and that "26" is an "Age" is enabled in the S3DB KOS. S3DB's use of named graphs to describe the domain enables updates to the domain without affecting the consistency of its instantiation - in the example above, modifying "hasAge" with "hasAgeInYears" will not affect queries that have already been assembled using that property.

**Figure 1:**
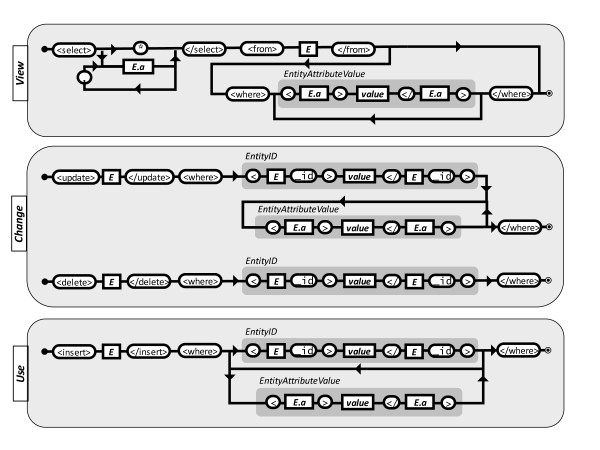
**S3QL language specification using rail diagrams**. Rail diagrams are read from the left to the right - any string that can be composed following these diagrams is a valid S3QL query. Valid forms of *E *and *E.a *will vary according to the Core Model used in the KOS. For example, if the S3DB Core model is used, any entity in figure 2 can be used in place of *E*; upon choice of *E, E.a *is any attribute that can be attained by following a line from *E*.

**Figure 2:**
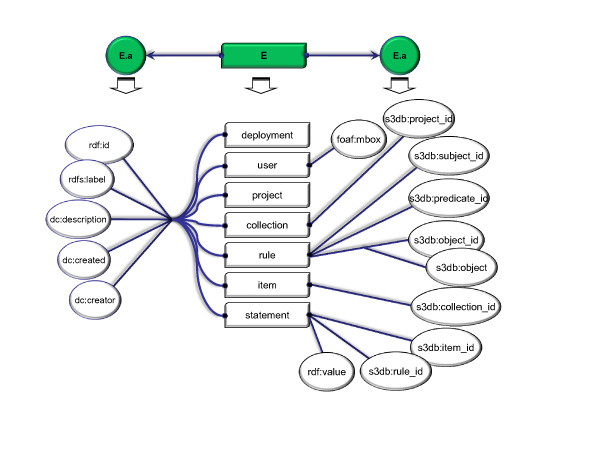
**Entities in the S3DB Core Model and its attributes**. A minimal set of common attributes was defined (left) for each of the S3DB entities using RDF Schema (rdfs) and Dublin core (dc) terminology - these are *rdf:id, rdfs:label, dc:description, dc:created *and *dc:creator*. Other attributes, which are specific to each of the S3DB entities (right), such as *foaf:mbox *for the entity *User *or *s3db:project_id *for the entity *Collection *reflect the *s3db:relationships *described above and formalized in the S3DB conceptual model (figure 3).

**Figure 3:**
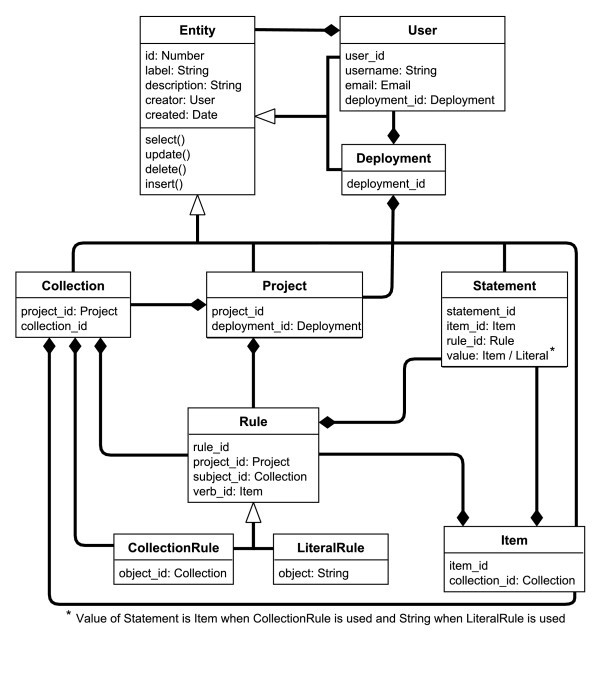
**The S3DB conceptual model**. Five attributes (id, label, description, creator and created) and four methods (select, update, insert and delete) are common to all S3DB entities. In the current S3QL implementation, the label and description attributes are defined by the submitter of the data, whereas the id, created and creator attributes are automatically assigned by the system. Other dependencies were devised to comply with the definition of *s3db:relationships*.

In the S3DB core [40], a meta-model for this data is also created with the specific objective of enabling propagation of operations, such as permission assignments, between the domain description and the data itself, described in the following section (see Figures 1, 2 and 3). In the example above, the two triples are respectively assigned to entities of type *s3db:rule *and *s3db:statement *where indexes "Person" is identified by a named graph of type *s3db:collection *and "John" is identified by a named graph of type *s3db:item*. The S3DB Core specifies three other entities which are specifically devised to enable knowledge organization and operator propagation: *s3db:project *entails a list of *s3db:rule *and *s3db:collection *and are typically applied in domain contextualization; *s3db:deployment *corresponds to the physical location of an S3DB system (its URL) and *s3db:user *is the subject of permission assignment operations. It is worth noting that, by making use of S3DB entities, blank nodes are avoided by assigning a unique alphanumeric identifier to every instance of an S3DB entity. The S3DB entities can also be identified using the first letter of their names, D, P, R, C, I, S or U, which will be used in subsequent examples to indicate, respectively, *s3db:deployment, s3db:project, s3db:rule, s3db:collection, s3db:item, s3db:statement *or *s3db:user*.

### Operators for Permission Control

The second key feature that makes S3DB appropriate for controlled management operations is support for permission control embedded in its core model. As described above and in [40], the hierarchy of permissions to view/edit entities in S3DB is modelled by an adjacency matrix, which is used as a transition matrix in the propagation of permission states. For a walkthrough of the propagation mechanism, see additional file 1. The *s3db:operator *states applied to the S3DB transition matrix modulate propagation by three core functions - merge, migrate and percolate. This behaviour for propagation of permission is described in detail in equation 5 of [40] and is reproduced here in Equation 1. The S3DB transition matrix (T) is defined by 12 *s3db:relationships *describing dependencies and inference rules between entities of the S3DB core model. The operator state vector (f) is used as the predicate of a triple established between an *s3db:user *and an entity of the S3DB core model. The JavaScript application at [51] can also be used to attempt this set of propagation behaviours for *s3db:operators *both on the S3DB transition matrix or with alternative adjacency matrixes.(1)

The *s3db:operators *[40] have a scope and applicability in linked data beyond permission management. In S3QL we define three operator types for controlled management operations: for each of the rights to view, change/edit or use instances of S3DB entities. The format used to assign permission was defined as a three character string, where each operator occupies respectively the first, second or third positions and may assume value N, S or Y according to the level of permission intended: no permission (N), permission limited to the creator of the resource (S) or full permission (Y). For example, the permission assignment "YSN" specifies complete permission to view (Y) the subject entity, partial permission to change it (S) and no permission to use it (N). States may be defined as dominant, by use of uppercase (Y, N or S) or recessive, by use of lowercase characters (y, n or s). Dominant and recessive permissions are used to decide on the outcome of multiple permissions converging on the same entity (as detailed in [40]). Missing permission states, indicated by the dash character '-' (which has no lower or upper case) are also allowed, as well as a mechanism to succinctly specify transitions with variable memory length (*l *in equation 1). The propagation of permissions in the S3DB Core Model ensures that for every entity and every user, two types of permission are defined: the assigned permission, or the permission state assigned directly to a user in an entity, and the effective permission, which is the result of the propagation of *s3db:operators*.

### Components of a Distributed System

One of the requirements for RDF-based knowledge management ecosystems is the availability of queries spanning across multiple SPARQL endpoints. Automation of distributed queries in systems supporting permission control, such as S3DB, is challenged by user authentication. In S3QL, we propose addressing this through delegation to authentication authorities. As a result, a user (or usage), can be identified by a URI that is independent of the authorities that validate it. Whenever possible, it is recommended that authentication credentials be protected by use of OAuth [53].

Use of URIs and Internationalized Resource Identifiers (IRIs) to identify data elements is one of the core principles of Linked Data. However, many programming environments cannot easily handle URIs as element identifiers. Problems range from decreased processing speed to a need for encoding the URIs in web service exchanges. As an anticipation for that class of problems, the URIs for entities in S3DB are interchangeable with alphanumeric identifiers formulated as the concatenation of one of D, U, P, C, R, I or S (referring to S3DB entities described in The S3DB Knowledge Organizational Model) identifying the entity and a unique number. As an example, for a deployment located at URL http://q.s3db.org/demo, the alphanumeric P126 is resolvable to an entity of type Project with URI http://q.s3db.org/s3dbdemo/P126. To facilitate exchange of URI in distinct deployments, the URI above could also be specified as D282:P126, where "D282" is the alphanumeric identifier of the S3DB deployment located at URL http://q.s3db.org/demo. Every *s3db:deployment *is identified by a named graph in the form D[number]; for completeness, metadata pertaining to each *s3db:deployment*, such as the corresponding URL, is described using the vocabulary of interlinked datasets (VoiD) [19] and shared through a root location.

### Availability and Documentation

The specification of the S3QL language has been made available at http://link.s3db.org/specs and one example of the output RDF is available at http://link.s3db.org/example. S3QL has been implemented through a REST application programming interface (API) for the S3DB prototype, which is publicly available at http://s3db.org. Both the prototype and its API were developed in PHP with MySQL or PostgreSQL for data storage. Documentation about the S3DB implementation of S3QL as an API can be found at http://link.s3db.org/docs. S3QL queries may be tested at the demo implementation at http://link.s3db.org/s3qldemo and a translator for the compact notation is available at http://link.s3db.org/translate.

## Results

### S3QL Syntax

S3QL is a domain specific language devised for facilitating management operations such as "insert", "update" or "delete" using entities of a Linked Data KOS such as the S3DB core model described above. Its syntax, however, is loosely tied to the S3DB Core Model, and can easily be applied to a set of KOS' core models in which S3DB is included. The complete syntax of S3QL in its XML (eXtended Markup Language) flavour is represented in the railroad diagram of Figure 1. The S3QL syntax includes three elements: the description of the operation, the target entity and the input parameters. Four basic operation descriptions were deemed necessary to fully support read/write operations: select, insert, update and delete. The action of these operations mimic those of the structured query language (SQL) and target instances of entities (*E*) defined in the core model. Input parameters include the set of attributes defined for each of the entities either in the alphanumeric form associated with entity instances (*EntityId*) or in the form of *EntityAttributeValue (E.a)*. The values for *E.a *are determined upon choice of E - for an example using S3DB entities and attributes see Figure 2: E may be replaced with any of the entities defined in the S3DB Core Model (*Deployment, User, Project, Collection, Rule, Item *or *Statement*); upon choice of *E*, valid forms of *E.a *include any of the attributes defined for *E *(e.g. rdf:id, rdfs:label, rdfs:comment). A table summarizing all available operations, targets and input parameters is made available at http://link.s3db.org/specs. The formal S3QL syntax is completed by enclosing the outcome of one of the diagrams on Figure 1 with the <S3QL> tag. For example, the following XML structure is a valid S3QL query for an operation of type insert where the target is the S3DB entity Project and the input parameter, formulated as EntityAtributeValue is "label = Test":

<S3QL>

  <insert>project</insert>

  <where>

   <label>Test</label>

  </where>

</S3QL>

The set of 12 *s3db:relationships *(see Table one of [40]) in the S3DB Model determine the organizational dependencies of S3DB entities. For example *s3db:PC *is the *s3db:relationship *that specifies a dependency between an instance of a *Collection *(C) and an instance of a *Project *(P) (Figure 3). The S3QL syntax fulfils this constraint by assigning *project_id *(the identifier of an S3DB *Project*) as an attribute of a *Collection*. In this description of attributes associated with the S3DB core model we make use of the assumption, as in other KOSs and in the Linked Data in general, that there is no restriction to adding relationships beyond those described here. S3QL was identified as the minimal representation to interoperate with the S3DB core model and therefore only those relationships are explored in this report.

The syntax diagram in Figure 1 generates XML, a standard widely used in web service implementations. That alternative often results in verbose queries that could easily be assembled from more compact notations. One example to consider is the form: *action (E | E.a = value)*. Here the symbol '|' should be interpreted, as in Bayesian inference, as a condition and be read "given that". The letter "*E*" corresponds to the first letter of any S3DB entity (D, P, R, C, I, S or U) and *E.a *is any of its attributes as described in Figure 2. In this example, the query *insert(P | label = test) *is equivalent to the example query above. That particular variant is also accepted by the S3DB prototype and a converter for this syntax into complete S3QL/XML syntax was made available at http://link.s3db.org/translate. For further compactness of this alternative formulation, entity identifiers used as parameters may be replaced with its corresponding alphanumeric identifiers - for example *project_id = 156 *may be replaced with *P156*. This alternative notation will be used in the subsequent examples.

### S3QL Permission Control

Permission states are assigned using an S3QL query such as *insert(U| U1,P157,permission_level = ysn)*, which includes the action insert, the target entity User and three input parameters: identifier of the User (U1), identifier of the entity (P157) and permission assignment (ysn). Effectively, this will result in the creation of the triple *[:U1: ysn: P157]*, where the subject is of type *s3db:user*, the predicate is of type *s3db:operator *and the object is of type *s3db:project*. The inclusion of this triple in a dataset will modulate the type of management operation that a user may perform. As described in the Methods section, each position in the permission assignment operator (ysn) encodes, respectively, for permission to "view", "change" or "use" the object entity. Values y, s and n indicate, respectively, that the user has full permission to view it (y), permission to change its metadata only if he was the creator of the entity (s) and no permission to insert (n) child entities. Each S3QL operation is therefore tightly woven to each of the three operators: select is controlled by "view"; update and delete are controlled by "change" and insert is controlled by "use" (shaded areas in Figure 1). The 'use' operator encodes for the ability of a user to create new relationships with the target entity, which is defined separately from the right to "change" it. For example, in the case of a user (U1) being granted "y" as the effective permission to "change" an *s3db:rule*, then the metadata describing it may be altered. If, however, that same user is granted permission "n" to 'use' that same Rule, she is prevented from creating Statements using that Rule. Although "use" may be interpreted as being equivalent to "insert" or "append" in other systems, we have chosen to separate the terms describing the operator "use" from the S3QL action "insert". The permission assigned at the dataset level will then propagate in the S3DB transition matrix following the behaviour formalized in equation 1, therefore avoiding the need to assign permission to every user on every entity. It is worth noting that the DSL presented here is extensible beyond the 4 management actions (select, insert, update, delete) described. The *s3db:operators *that control permission on these actions are also extensible beyond "view", "change" and "use" and different implementations may support alternative states.

**Figure 4:**
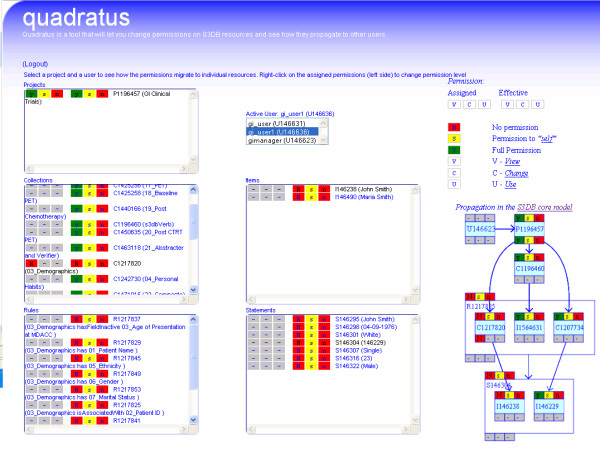
**Quadratus, an interface to illustrate S3QL's permission control mechanism and its effect on S3QL queries**. *Projects, Collections, Rules, Items *and *Statements *associated with GI Clinical Trials *Project *are displayed with effective and assigned permissions. *Collections *and *Rules *retrieved using S3QL queries *select(**C**|P1196457) *and *select(**R**| P1196457) *inherit the permission *assignment *in the *Project *"ysn"; *assigned *permission "N--" in *Collection *"Demographics" results in the *effective *permission of "Nsn" inherited by all *Rules *and *Items *that have a relationship with that *Collection*, effectively preventing gi_user1 from accessing its data. The directed labelled graph of the propagation resolution is displayed on the right side of the application illustrating the propagation mechanism.

The permission control behaviour for S3QL operations can be illustrated through the use of the Quadratus, an application available at http://q.s3db.org/quadratus that can be pointed at any S3DB deployment to assign permission states on S3DB entities to different users (Figure 4). Other use case scenarios are also explored in the S3QL specification at http://link.s3db.org/specs.

### Global Dereferencing for Distributed Queries

**Figure 5:**
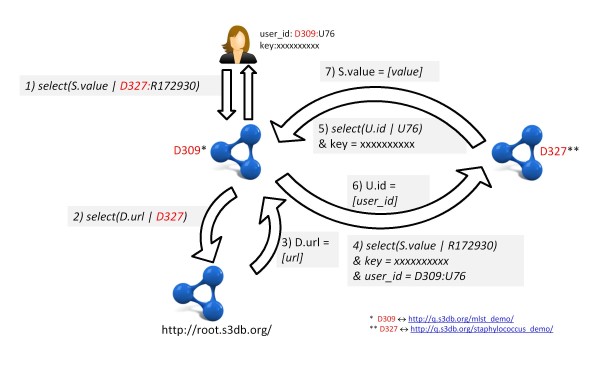
**The global S3QL dereferencing system**. User *U78 *of deployment *D309 *issues a command to request all entities of type *Statement *where the attribute *Rule*_*id *corresponds to the value *D327:R172930 *through S3QL operation *select(S.value|D327:R172930) *(1). If the URL corresponding to *deployment D327 *is not cached locally or has not been validated in the past 24 h, a query is issued and executed at the root deployment to retrieve the corresponding URL *select(D.url|D327) *(2,3). Once the URL is returned, query (1) is re-issued as *select(S.value| R172930) *and executed at the URL for *D327 *(4). To validate the user, deployment *D327 *issues the command *select(U.id|U76) *at *D309 *using the key provided (5,6) and returns the data only if *U.id *matches the value for *user_id *(7).

A simple dereferencing system was devised for S3DB identifiers that relies on the identification of root deployments, i.e. S3DB systems where alphanumeric identifiers for S3DB deployments can be dereferenced to URL. This simple mechanism enables complex transactions of controlled data. For an example of this behaviour see Figure 5, where the S3DB UID *D327:R172930*, identifying an entity of type Rule (R172930) available in deployment D327 is being request by a user registered in deployment D309. In order to retrieve the requested data, the URL of deployment D327 must first be resolved at a root deployment such as, for example, http://root.s3db.org.

The dereferencing mechanism is also applicable in more complex cases where the root of two deployments sharing data is not the same. Prepending the deployment identifier of the root to the UIDs such as, for example, *D1016666:D327:R172930*, where D1016666 identifies the root deployment, would result in recursive URL resolution steps such as *select(D.url| D1016666) *prior to step 4 in Figure 5. This mechanism avoids broken links when S3DB deployments are moved to different URLs by enabling deployment metadata to be updated securely at the root using a public/private key encryption system.

### Implementation and benchmarking

In the current prototype implementation, S3QL is submitted to S3DB deployments using either a GET or POST request and may include an optional authentication token (key). The REST specification [54] suggest separate HTTP methods according to the intention of the operation: often, "GET" is used to retrieve data, "PUT" is used to submit data, "POST" is used to update data and "DELETE" is used to remove data. There are, however, many programming environments that implement only the REST "GET" method, including many popular computational statistics programming frameworks such as R and Matlab. Therefore, in order to fully explore the integrative potential of this read/write semantic web service, and to support operations beyond the 4 implemented, the S3DB prototype implementation of S3QL supports the "GET" method for all S3QL operations, with the parameters of the S3QL call appended to the URL. One drawback of relying on GET is the limits imposed by the browser on URL length. To address this potential problem, the S3DB prototype also supports the use of "POST" for S3QL calls.

Two further challenges needed to be addressed in the prototypical implementation of S3QL: 1) the need for a centralized root location to support dereferencing of deployment URI when the condensed version is used (e.g. D282) and 2) distributed queries on REST systems required users to authenticate in multiple KOSs. The first challenge was addressed by configuring an S3DB deployment as the root location, available at http://root.s3db.org. Deployment metadata is submitted to this root deployment at configuration time using S3QL; data pertaining to each deployment can therefore be dereferenced to a URL using http://root.s3db.org/D[numeric]. The option to refer to another root deployment than the default is possible during installation. To avoid overloading these root deployments with too many requests, a local 24 hour cache of all accessed deployments is kept in each S3DB deployment using the same strategy; if the URL is cached, it will not be requested from the root deployment. To address the second challenge, each *s3db:deployment *can store any number of authentication services supporting HTTP, FTP or LDAP protocols. Once the user is authenticated, temporary surrogate tokens are issued with each query. When coupled with the user identifier in the format of a URI, these tokens effectively identify the user performing the query regardless of the S3DB deployment where the query is requested.

Screencasts illustrating processing time of data manipulation using S3QL are available at http://www.youtube.com/watch?v=2KZC6kI609s and http://www.youtube.com/watch?v=FJSYLCwBaPI.

## Discussion

One of the major concerns in making use of Linked Data to improve health care and life sciences research is the need to ensure both the availability of contextual information about experimental datasets and the ability to protect the privacy of certain data elements which may identify an individual patient. Domain specific languages (DSL) can ease the task of managing the contextual descriptors that would be necessary to implement permission control in RDF and, by doing so, could greatly accelerate the rate of adoption of Linked Data formalisms in the life sciences communities to improve scientific discovery. We have described S3QL, a DSL to perform read/write operations on entities of the S3DB Core Model. S3QL attempts to address the requirements in linking Life Sciences datasets including both publishable and un-publishable data elements by 1) including contextual descriptors for every submitted data element and 2) making use of those descriptors to ensure permission control managed by the data experts themselves. This avoids the need to break a consolidated dataset into its public and private parts when the results are acceptable for publication.

Applying S3QL to the S3DB Core Model in a prototypical application benefited from the definition of loosely defined boundaries for RDF data that enabled propagation of permission while avoiding the need to document a relationship for each data instance, individually, and for each user. The assembly of SPARQL queries is also facilitated by the identification of domain triples using named graphs, from the data itself [41] and can be illustrated in the application at [55], where a subset of S3QL can be readily converted into the W3C standard SPARQL. Although the prototypical implementation of S3QL fits the definition of an API for S3DB systems, it is immediately apparent that the same notation could be easily and intuitively extended to other KOS' core models. For example, pointing the tool at http://q.s3db.org/translate to a JavaScript Object Notation (JSON) representation of the SKOS core model (skos.js) instead of the default S3DB core model (s3db.js) results in a valid S3QL syntax that could easily be applied as an API for SKOS based systems, as illustrated by applying the example query "*select(C|prefLabel = animal)*" using http://link.s3db.org/translate?core=skos.js&query=select(C|prefLabel=animal) to retrieve SKOS concepts labelled "animal". The progress of adoption for life sciences application developers can be further smoothed by complete reliance on the REST protocol for data exchange and the availability of widely used formats such as JSON, XML or RDF/turtle.

The applicability of S3QL to life sciences domains is illustrated here with three case studies: 1) in the domain of clinical trials, a project [42] that requires collaboration between departments with different interests; 2) in the domain of the cancer genome atlas (TCGA) project, a multi-institutional effort that requires multiple authentication mechanism and sharing of data among multiple institutions [41] and 3) in the domain of molecular epidemiology, a project where non-public data stored in an S3DB deployment needs to be statistically integrated with data from a public repository. All described use cases shared one considerable requirement - the ability to include, in the same dataset, both published and unpublished results. As such, they required both the annotation of contextual descriptors of the data, enabled by S3QL, and the availability of controlled permission propagation, enabled by the S3DB model transition matrix. Future work in this effort may include the application of S3QL in a Knowledge Organization System based on SKOS terminology and the definition of a transition matrix for SKOS to enable controlled permission propagation.

### Gastro-intestinal Clinical Trials Use Case

**Figure 6:**
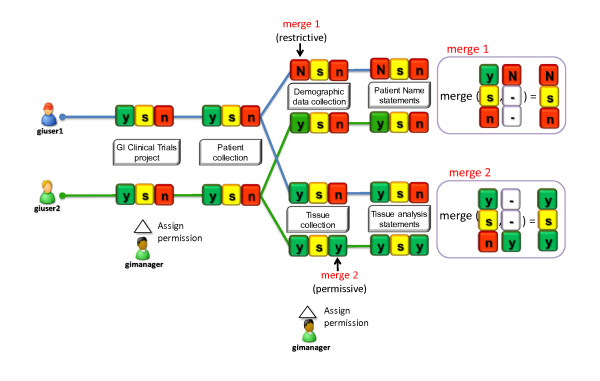
**Two use cases of permission propagation**. Two users are granted full permission to *view *GI Clinical Trials *Project *("y"), however none of them can add new data ("n") nor edit existing data unless they were its original creator ("s"). One of the users (giuser1) is prevented from accessing any data with demographic elements such as, for example, the names of the Patients. In this case, an uppercase "N" is assigned in the right to *view *the *Collection *DemographicData, which will be merged with the inherited "y" to produce an effective permission level of "N" for the right to *view *(merge 1). For the second user (giuser2), permission is granted to *use *the *Collection *TissueData, indicating that the user can create new instances in that *Collection *(merge 2).

As part of collaboration with the Department of Gastrointestinal Medical Oncology at The University of Texas M.D. Anderson Cancer Center, an S3DB deployment was configured to host data from gastro-intestinal (GI) clinical trials. A schema was developed using S3DB Collections and Rules and S3QL insert queries were used to submit data elements as Items and Statements (see Figure 6). Two permission propagation examples are illustrated, one of a restrictive nature and the other permissive, which can also be explored at http://q.s3db.org/quadratus (Figure 4). In this example, the simple mechanisms of propagation defined for S3QL support the complex social interaction that requires a fraction of the dataset to be shared with certain users but not with others. Contextual usage is therefore a function of the attributes of the data itself (e.g. its creator) and the user identification token that is submitted with every read/write operation.

### The Cancer Genome Atlas Use Case

The cancer genome atlas (TCGA) is a pilot project to characterize several types of cancer by sequencing and genetically characterizing tumours for over 500 patients throughout multiple institutions. S3QL was used in this case study to produce an infrastructure that exposes the public portion of the TCGA datasets as a SPARQL endpoint [41]. This was possible because SPARQL is entailed by S3QL but not the opposite. Specifically, SPARQL queries can be serialized to S3QL but the opposite is not always possible, particularly as regards write and access control operations. The structure of the S3DB Core Model which explicitly distinguishes domain from instantiation enables SPARQL query patterns, such as *?Patient: R390 ?cancerType *to be readily serialized into its S3QL equivalent *select(S|R390)*. Although this will not be further explored in this discussion, it is worth noting that the availability of this serialization allows for an intuitive syntax of SPARQL queries by patterning them on the description of the user-defined domain Rule, such as, *"Patient hasCancerType cancerType"*.

### A Molecular Epidemiology Use Case

**Figure 7:**
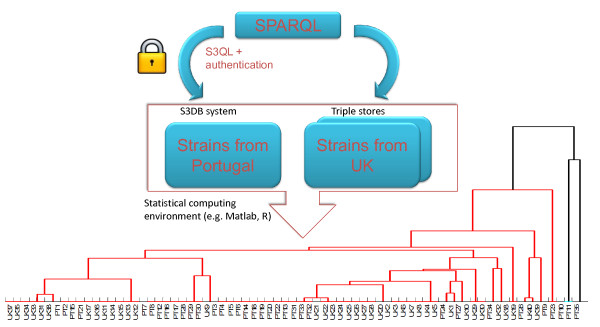
**Workflow for the creation of a hierarchical cluster of MLST profiles from strains collected in Portugal and in the UK**. Two sources are chosen to perform the MLST profile assembly - an S3DB *deployment*, which holds the ITQB *Staphylococcus *database, and the SPARQL endpoint configured to host the *Staphylococcus aureus *MLST database. Although data in the MLST SPARQL endpoint is publicly available, to access data in S3DB, the user needs to provide an authentication token and a *user_id *as well as assemble the S3QL queries to retrieve the data [*select(S|R172930) *and *select(S|R167271)*], which may also be formulated as SPARQL. A data structure is assembled that can finally be analysed using hierarchical clustering methods such as the ones made available by Matlab. The complete MLST dataset used to obtain the graph is provided in additional file 2.

In this example, SPARQL was serialized to S3QL to support a computational statistics application. As a first step, an S3DB data store was deployed using S3QL to manage molecular epidemiology data related to strains of *Staphylococcus aureus *bacteria collected at the Instituto de Tecnologia Química e Biológica (ITQB), in Portugal. Specifically, the ITQB *Staphylococcus *reference database was devised with a purpose of managing Multilocus Sequence Typing (MLST) data, a typing method used to track the molecular epidemiology of infectious bacteria [54-57]. As a second step, we downloaded the public *Staphylococcus aureus *MLST profiles database at http://www.mlst.net and made it available through a SPARQL endpoint at http://q.s3db.org/mlst_sparql/endpoint.php. The process of integration of MLST profiles from the ITQB Staphylococcus database with the publicly available MLST profiles is illustrated in Figure 7. In this example, a federated SPARQL query is assembled to access both MLST sources; data stored in the S3DB deployment is retrieved by serializing the SPARQL query into S3QL and providing an authentication token to identify the user, as described in Figure 3. The assembled graph resulting from the federated SPARQL query (see additional file 2), can be imported into a statistical computing environment such as Matlab (Mathworks Inc). Using this methodology, it was possible to cluster strains from two different data sources with very different authentication requirements. The observation that some Portuguese strains (PT1, PT2, PT15 and PT21) that are not publicly shared cluster together with a group of public UK strains (UK17, UK16, UK11, UK14, UK15, UK13, UK12) and therefore may share a common ancestor is an observation enabled by the data integrated through S3QL.

## Conclusions

Life sciences applications are set to greatly benefit from coupling Semantic Web Linked Data standards and KOSs. In the current report we illustrate data models from life sciences domains weaved using the S3DB knowledge organization system. In line with the requirements for the emergence of evolvable "social machines", different perspectives on the data are made possible by a permission propagation mechanism controlled by contextual attributes of data elements such as its creator. The operation of the S3DB KOS is mediated by the S3QL protocol described in this report, which exposes its Application Programming Interface for viewing, inserting, updating and removing data elements. Because S3QL is implemented with a distributed architecture where URIs can be dereferenced into multiple S3DB deployments, domain experts can share data on their own deployments with users of other systems, without the need for local accounts. Therefore, S3QL's fine grained permission control defined as instances of *s3db:operators *enables domain experts to clearly specify the degree of permission that a user should have on a resource and how that permission should propagate in a distributed infrastructure. This is in contrast to the conventional approach of delegating permission management to the point of access. In the current SPARQL specification extension various data sources can be queried simultaneously or sequentially. There is still no accepted convention for tying a query pattern to an authenticated user, probably because SPARQL engines would have no use for that information as most have been created in a context of Linked Open Data efforts. The critical limitation in applying this solution for Health Care and Life Sciences is the ability to make use of contextual information to determine both the level of trust on the data and to enable controlled access to elements in a dataset without breaking it and storing it in multiple systems. To address this requirement, S3QL was fitted with distributed control operational features that follow design criteria found desirable for biomedical applications. S3QL is not unique in its class, for example the linked data API, which is being used by data.gov.uk is an alternative DSL to manage linked data [58]. However, we believe that S3QL is closer to the technologies currently used by application developers and therefore may provide a more suitable middle layer between linked data formalisms and application development. It is argued that these features may assist, and anticipate, future extensions of semantic web provenance control formalisms.

## Competing interests

The authors declare that they have no competing interests.

## Authors' contributions

HFD developed the S3QL domain specific language, implemented it in the S3DB prototype, validated it with examples and wrote the manuscript. MCC, RS, MM, HL, RF, WM and JSA tested and validated the language with examples and made suggestions which lead to its improvement. All authors read and approved the final manuscript.

## Supplementary Material

Additional file 1 **Walkthrough of S3DB's permission propagation: merge, migrate and percolate**. Click here for file

Additional file 2 **Matrix of MLST profiles in Portugal and the United Kingdom**. Click here for file
