# Supplementary material for: S3QL: A distributed domain specific language for controlled semantic integration of life sciences data
Source: BMC Bioinformatics. 2011 Jul 14;12:285. doi: 10.1186/1471-2105-12-285 (PMC3155508; doi:10.1186/1471-2105-12-285)

# A walkthrough of S3DB’s permission propagation: merge, migrate and percolate

We present here detailed example use cases of percolating permission from an entity of type *s3db:project* to instances of type *s3db:collection*, *s3db:rule, s3db:item and s3db:statement*.

Assume the following S3DB relationship describing a project (library), two collections (books and notes), a property for a collection (books_have_name), one instance of the collection books (book[i]) and the assertion of a property for book[i] (book name).

<P_library> rdf:type s3db:project; s3db:PC <C_books> ;

s3db:PR <R_books_have_name> .

<C_books> rdf:type s3db:colection;

s3db:CI <I_book[i]> ;

s3db:Rsubject <R_book_ name> .

<C_notes> rdf:type s3db:colection .

<R_book_ name> rdf:type s3db:rule .

<I_book[i]> rdf:type s3db:item;

s3db:IS <S_book[i].Name> .

<S_book[i].Name> rdf:type s3db:statement;

rdf:value “Gone with the Wind” .

Assume also the S3DB transition matrix (T) where each row and each column represent one S3DB entity as part of the set of *s3db:entities* identified in [40]. The binary values in the matrix identify possible existing arcs between the *s3db:entities*.

*S3DB transition matrix (T) =*
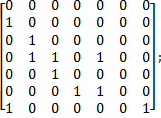
 *S3DB entities (E) =*
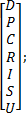


Making use of *s3db:operators*, a permission assignment example corresponds to assigning y (yes) on “view” and n (no) on modify to a user on a *s3db:project*. Since modify can be separately expressed to “edit” an entity description and to “use” it in the assertion of new instances, the assigned permission state can be formulated as:

*f_P_* = “ynn”

In the equality above, “y” indicates permission to view, the first “n” indicates lack of permission to “edit” and the second “n” indicated lack of permission to “use” an entity for creating novel assertions. The RDF assertion equivalent to the equality above corresponds to (equation 1 in [40]):

*<U1> <permission:ynn> <* P_library *> .*

By default, permission assigned to the project will propagate unaltered to every dependency of the project, i.e. if no other permission assignment is specified, the permission propagation will behave according to equation 5 in [40]:

E_0_ *=*
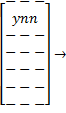
 *T xE_k_=*
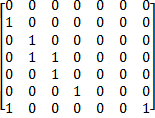

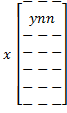
 = *E_k+1_*; *TxE_k+1_* = .... =
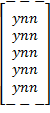
;

Resulting in the asserted permission states:

***<U1> <permission:ynn> <* C_books *> , <* C_notes *>,* <R_book_ age> , <I_book[i]> , <S_book[i].Name> .**

Assume now that the owner of project <P_library> wishes to specify a special permission assignment only to collection <C_books>, which should not affect any other collections in the same project. One example of such a case would be the ability for other users to add new instances of <C_book> without, however, granting permission to change any other collection in the project.

The project owner may define a new permission for the collection using:

*f_c_* = “-sy”

*Or:*

*<U1> <permission:-sy> <* C_books *> .*

In such cases, where there is more than 1 permission assignment for the same entity, permission states will merge. Merging rules determine that uppercase permission states (Y/S/N) always have priority over lowercase permission states (y/s/n), which in turn have priority over undefined permission states (-). Permissions also have an order, which for convienience is assumed to correspond to its position in the Latin alphabet: P(13) = n; P(18) = s and P(22) = y. According to equation 2 in [40], when the states to be merged are uppercase, then the most restrictive permission should be used (*i*_|A≠null_ = min(A) ). If, however, no uppercase states are defined, the most permissive permission should be used (*i*_|A=null_ = max(a)).

Given that, in the example above, A = null, merging states will result in the following:

View: *merge({y, -}) → P(max(22, 0)) = P(22) = y;*

Edit: *merge({n, s}) → P(max(13, 18)) = P(18) = s; merge (“ynn”,”-sy”) = “ysy”*

Use: *merge({n, y}) → P(max(13, 22)) = P(22) = y;*

Note that merge will only affect resources downstream of <C_books>, therefore <C_notes> will inherit permission only from <P_library>.

A permission state vector will be propagated with merged states:

*f*_p_ *=*
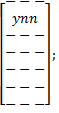
 *f*_c_ *=*
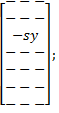
merge ( [*f*_C_, T x *f*_P_]) *=*
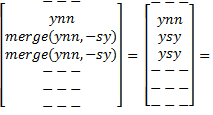
*…*
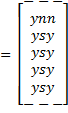


Effectively resulting in the permission assignments:

***<U1> <permission:ynn> <* P_library *> , <*C_notes*> .***

***<U1> <permission:ysy> <* C_books *> ,* <R_book_name> , <I_book[i]> , <S_book[i].Name> .**

Note that the specification of “s” in both <C_books> and <R_book_ age> will only allow the creator of those resources to modify their description. The “y” in the third slot will enable U1 to add new instances of <C_books> and <R_book_name> ; furthermore, “s” will propagate to instances <I_book[i]> created by U1, enabling him to modify such instances but none of the instances created by other users.

In another use case scenario, the creator of the project may wish to enable U1 to discover that the project <P_library> exists, but to prevent access to its contents:

*f_P_* = “ynnN”

*Or:*

*<U1> <permission:ynnN> <* P_library *> .*

Notice that the default length (l) of a permission assignment in *s3db:operators* is 3, i.e. permission to “view”, “edit” or “use” are encoded separately.

If, as in the case above, assigned permission states are defined where *l>3* any additional assignments will be migrated prior to percolation (equation 5 in [40]):

*l* = 3 -> migrate(“ynn”, 3) = f = “ynn”

*l* > 3 -> migrate(“ynnN”, 3) = migrate(“N”) = “NNN”

Notice that a final permission state must always have *l=3* ; when only one is specified for any given entity, migrate allows for the single assignment to span the several operators up to *l*.

Given that, in the example above, A ≠ null, merging states will result in the following:

View: *merge({y, N}) = N;*

Edit: *merge({n,N}) = N; merge (“ynn”,”NNN”) = “NNN”*

Use: *merge({n, N}) = N;*

The permission state vector is propagated with the following result:

E_0_ *=*
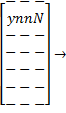
merge ( [E_k_, migrate (T xE_k_)]) *=*
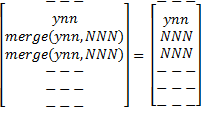
*= ... =*
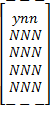


Resulting in the permission state statements:

***<U1> <permission:ynn> <*P_library*> .***

***<U1> <permission:NNN> <*C_books*> , <*C_notes*> , <*R_book_ age*> , <*I_book[i]*> , <* S_book[i].Name*> .***

The three functions described here – merge, migrate and percolate - have been made available both as JavaScript and Matlab libraries at <http://s3db-operator.googlecode.com/hg> and can be tested at <http://s3db-operator.googlecode.com/hg/propagation.html>:


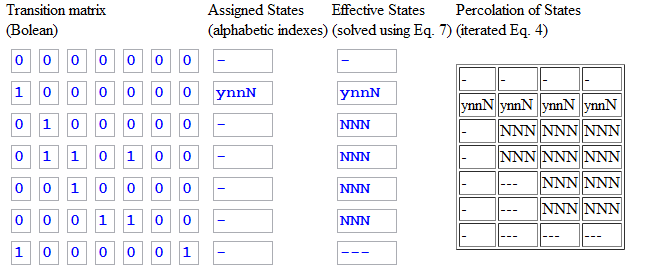

Supplement: Additional file 1 — Walkthrough of S3DB's permission propagation: merge, migrate and percolate. [file 1471-2105-12-285-S1.DOC]
